# Very-long-term efficacy of bioresorbable vascular scaffolds

**DOI:** 10.1007/s12471-017-1041-9

**Published:** 2017-10-05

**Authors:** R. Shah

**Affiliations:** Section of Cardiology, University of Tennessee, School of Medicine, Memphis, TN USA

To the Editor,

I read with great interest Elias and colleagues’ recent meta-analysis in which 2‑year follow-up data were used for all trials with the exception of ABSORB II [1]. However, sensitivity analysis using 2‑year follow-up data from ABSORB II does not change their summary result for target lesion failure (TLF) (RR 1.31; 95% CI 1.08–1.58; *p* = 0.004). Thus, based on this meta-analysis, we can conclude that bioresorbable vascular scaffolds (BVSs) are associated with worse safety and efficacy outcomes up to 2 years after implantation; however, it is not clear if this is true beyond 2 years.

Since the publication of this work, two randomised controlled trials (RCTs) in addition to ABSORB II have reported 3‑year results [2, 3]. In ABSORB-Japan, only one scaffold thrombosis occurred (0.4%) in the BVS group between years 2 and 3 [3]. The TLF rate was exactly the same (1.6%) for both the BVS and metallic stent groups. In ABSORB-China, no stent thromboses occurred in either group between years 2 and 3 [2]. A landmark meta-analysis (using TLF between years 2 and 3) of these three trials suggests no statistically significant difference in TLF between the BVS and metallic stent groups (RR 1.78; 95% CI 0.68–4.62; *p* = 0.237).

These new findings suggest that efficacy problems with BVSs might recede 2 years post-implantation. While these findings are encouraging, they are not confirmatory; to be so, continued long-term follow-up of all relevant RCTs is needed.
